# Animal Health and Welfare Issues Facing Organic Production Systems

**DOI:** 10.3390/ani3041021

**Published:** 2013-10-31

**Authors:** Mhairi A. Sutherland, Jim Webster, Ian Sutherland

**Affiliations:** 1 Innovative Farms Systems, AgResearch Ltd, Ruakura Research Centre, Hamilton 3240, New Zealand; E-Mail: jim.webster@agresearch.co.nz; 2 Animal Productivity, AgResearch Ltd, Grasslands, Palmerston North 4442, New Zealand; E-Mail: ian.sutherland@agresearch.co.nz

**Keywords:** animal welfare, health, organics, standards

## Abstract

**Simple Summary:**

The demand for organically grown, animal derived produce is increasing due to a growing desire for consumer products that have minimal chemical inputs and high animal welfare standards. Evaluation of the scientific literature suggests that a major challenge facing organic animal production systems is the management and treatment of health-related issues. However, implementation of effective management practices can help organic animal producers achieve and maintain high standards of health and welfare, which is necessary to assure consumers that organic animal-based food and fibre has not only been produced with minimal or no chemical input, but under high standards of animal welfare.

**Abstract:**

The demand for organically-grown produce is increasing worldwide, with one of the drivers being an expectation among consumers that animals have been farmed to a high standard of animal welfare. This review evaluates whether this expectation is in fact being met, by describing the current level of science-based knowledge of animal health and welfare in organic systems. The primary welfare risk in organic production systems appears to be related to animal health. Organic farms use a combination of management practices, alternative and complementary remedies and convenional medicines to manage the health of their animals and in many cases these are at least as effective as management practices employed by non-organic producers. However, in contrast to non-organic systems, there is still a lack of scientifically evaluated, organically acceptable therapeutic treatments that organic animal producers can use when current management practices are not sufficient to maintain the health of their animals. The development of such treatments are necessary to assure consumers that organic animal-based food and fibre has not only been produced with minimal or no chemical input, but under high standards of animal welfare.

## 1. Introduction

The international demand for organic products has increased significantly in recent years [1], suggesting that the production of food and fibre with minimal or no chemical input is a desirable characteristic of organic farming systems. In addition to a lack of synthetic chemicals used in production processes, another expectation of consumers who purchase organic food is that animal welfare standards are higher in organic than in non-organic production systems [1]. The assumption of high welfare standards in organic systems could be considered counter-intuitive, given that many of the synthetic chemicals that are prohibited in organic systems are administered in non-organic systems to prevent or treat animal health concerns and reduce the potential for suffering. Does the presence of an organic label guarantee a higher standard of welfare, and are the perceived attributes real and able to be substantiated?

The International Federation of Organic Agricultural Movements (IFOAM) [2] defines organic agriculture as “a production system that sustains the health of soils, ecosystems and people. It relies on ecological processes, biodiversity and cycles adapted to local conditions, rather than the use of inputs with adverse effects. Organic agriculture combines tradition, innovation and science to benefit the shared environment and promote fair relationships and a good quality of life for all involved”. This basic definition does not include any statement directly related to either the health or welfare of farmed animals, and alone does not address consumer expectations. The IFOAM Principles of Organic Agriculture [3] do refer to the health and well-being of animals in the Principle of Health “Organic Agriculture should sustain and enhance the health of soil, plant, animal, human and planet as one and indivisible” and the Principle of Care “Practitioners of organic agriculture can enhance efficiency and increase productivity, but this should not be at the risk of jeopardizing health and well-being”. However, is there any robust evidence that this intent is being met by commonly-used organic farming practices? 

In order to determine whether the welfare of animals is being adequately addressed in organic systems, it is crucial to be able to properly assess welfare. This has proved difficult for many researchers due to the different value-laden concepts of welfare which have resulted in a variety of preferred approaches to welfare assessment [4]. Fraser *et al.* [5] recognised that there is a number of overlapping ethical concerns regarding the welfare of animals, based on the relative value that individuals attribute to different dimensions of an animal’s life. These are (1) that animals should be able to lead natural lives, (2) that animals should be free of intense and prolonged negative affective states, such as fear and pain and (3) that animals should function well in the sense of having satisfactory health and physiology. If all these concerns are to be properly evaluated, then there is a need for a multi-disciplinary approach capable of assessing biological function (e.g., health, production, malnutrition), affective states (e.g., fear, pain, emotions) and natural living (e.g., presence of natural behaviors) [5]. The approach taken in this review, therefore, is to assess animal welfare in organic systems using the categories of (1) biological function, (2) affective state and (3) natural living. The aim of this review is to investigate the available scientific literature on animal health and welfare in a number of different organic agriculture production systems, or sectors. The resulting information will identify where there is a need for a robust scientific approach to the evaluation of particular organic practices.

## 2. Biological Function

### 2.1. Dairy Farming

In organic, as well as in non-organic dairy herds, the most common and important health issues are related to mastitis, lameness and infertility [6], with mastitis being the most frequently recorded or quoted disease [7]. 

#### 2.1.1. Mastitis

Inflammation of the mammary gland, resulting in mastitis, is predominantly caused by bacterial infection and is the most common animal health problem recorded on organic dairy farms [8,9,10,11,12,13]. While there is limited information on the relative extent and/or severity of mastitis in comparison to non-organic systems, studies have reported that mastitis and/or somatic cell counts (SCC) are similar, lower or higher on organic than on non-organic farms [10,12,13,14,15,16,17]. The observation that a reduced prevalence is not found on recently converted organic farms [15] may suggest that it takes time for organic farmers to successfully manage mastitis.

A major caveat when evaluating the prevalence of mastitis in dairy cattle as reported in the scientific or veterinary literature is that only infections treated by a veterinarian are routinely recorded and reported [10,15]. On organic farms, where alternative management strategies or remedies for mastitis are normally administered by the farmer rather than a veterinarian, fewer treatment records and lower rates of reporting may occur [10,15]. One survey conducted in Norway established that organic farmers were less likely to call a veterinarian to treat health problems than their non-organic counterparts, except for cases of milk fever [12]. This potential discrepancy between actual and reported cases of mastitis on organic farms was highlighted in a study from Sweden [18], in which the incidence of clinical mastitis treated and recorded by veterinarians was 9.1% in organic and 14.7% in non-organic herds; this was in contrast to an incidence of 11.7% recorded by organic farmers themselves.

An indirect indicator of udder health and mastitis is somatic cell counts (SCC) [10,13,17], measured from either individual animals or from milk collected in the bulk tank. The reported SCC in milk from organic herds were similar [12,13,16,17] or higher [10,11] than non-organic dairy herds, even though the number of reported cases of clinical mastitis on organic farms is often reported as been lower [10,12]. Consistent with the pattern of clinical mastitis, farms that had recently converted to organics had a higher SCC than established organic farms [15]. In a comparative study in New Zealand, in which organic and non-organic herds were run side by side, but with each unit managed according to best practice for the system, bulk milk SCC was higher in the organic herd initially however the opposite was found in subsequent seasons [19].

There is no apparent, simple relationship between reduced chemical input and the incidence of mastitis, with large differences in the number of cases of mastitis and/or SCC often recorded between farms, regardless of whether they are managed as organic or non-organic systems [8,9,14]. The reasons for such differences could include management practices such as cow genetics, feeding regimes, herd size, herd age and the volume of milk production [12,15,17,20]. Another difference could relate to farmer attitudes, and the motivation behind managing a farm organically; for example, do the farmers have a real belief in the ideologies/principles of organics, or are they more financially motivated [15]. The importance of the management approach on organic farms was illustrated in a comparative New Zealand study in which SCC were higher from an organic unit until 2008, and then the opposite was found in subsequent seasons [19]. This coincided with changing the focus of management practices from maximising yield to minimising SCC [19]. Furthermore, European organic dairy farms involved in an advisory process aimed at improving animal health and welfare and reducing the use of medicines (Animal Health and Welfare Planning) demonstrated a reduction in the number of udder and metabolic treatments during a one year study period [21].

#### 2.1.2. Lameness

Lameness is a serious welfare issue for the dairy industry [22]. In Sweden, the incidence of hoof disorders did not differ between organic and non-organic herds [14], while the incidence of lameness and hock lesions was found to be lower on organic than non-organic farms in the United Kingdom [22,23]. By contrast, some organic farms evaluated in the study had a high prevalence of hock damage [23], suggesting that organic management practices do not necessarily ensure a higher standard of foot health. 

Lameness is a multi-factorial problem with a number of risk factors, including age at calving, housing systems, average milk yield, lactation number, time on pasture and claw length [22,24]. A lower overall incidence of hoof problems in organic herds could be related to factors such as delayed breeding, a longer summer grazing period [22] and the effect of a relatively high forage diet [8].

#### 2.1.3. Metabolic Diseases

Organic certification requires that animal diets have a relatively greater level of roughage and lower use of feed supplements and vitamins compared to non-organic enterprises [6]. It is possible that a relatively lower quality and/or restricted diet could place organically-managed animals at enhanced risk of metabolic diseases such as milk fever and ketosis.

The incidence of milk fever, caused by calcium depletion as a result of regular milking, did not differ between organic and non-organic herds in Sweden [14], while the incidence was lower on organic farms in Norway [10,12]. The risk of milk fever rises by 5% with each kilogram increase in peak daily milk production, therefore, the relatively lower level of milk production from organic herds is a potential explanation for the reduced incidence of milk fever [10]. Ketosis occurs following the accumulation of large quantities of ketone bodies in blood and tissue, leading to metabolic acidosis. When the incidence of ketosis was compared between organic and non-organic farms in Sweden, no difference was observed [14]. A comparative study between organic and non-organic dairy farms in the United Kingdom found a tendency for more cows on organic farms to have sub-clinical ketosis [25]. However, cows were treated by a veterinarian for ketosis less regularly on organic than on non-organic farms in Norway [10,12]. In this case, the incidence of ketosis was higher in non-organic than organic herds which had converted before 1990, but similar between non-organic herds and newly-converted organic herds [15]. As is the case with milk fever, a possible explanation for this lower incidence of ketosis on organic farms is relatively lower milk production. 

#### 2.1.4. Reproductive Health

Infertility is a major issue for dairy cattle, resulting in reduced productivity and accelerated culling of animals in both non-organic and organic systems [6]. A variety of conditions contribute to infertility, including placental retention, endometritis and abortion [9]. The incidence of retained placenta was reported as being lower on organic than non-organic farms by both Hamilton *et al*. [14,18] and Valle *et al*. [12]. Over an 8 year period, veterinary treatment for retained placenta was higher in non-organic than organic herds established prior to 1990, but was similar between non-organic herds and newly converted herds [15]. Importantly, reproductive efficiency of organically managed herds was lower than non-organically managed herds once milk yield, breeding season, service and parity were taken into account, particularly in cows bred during winter [24]. Fall *et al.* [26] reported that older non-organically managed cows had a longer time from calving-to-first service than older organically reared cows, but this was the only difference in reproductive performance that they found. Reksen *et al*. [24] suggested that the reduction in reproductive efficiency was due to the cows’ energy requirements not being fully met during winter.

#### 2.1.5. Summary

In summary, according to the scientific literature, and official reporting of animal health cases, the main health issues facing organic dairy farmers are similar to those reported by non-organic farms. However, it would be dangerous to rely on this body of evidence alone, given the almost certain differences in reporting from organic and non-organic enterprises. This is at least in part due to the predominant application of alternative, non-veterinary treatments by organic farmers. Nonetheless, management practices utilized by organic dairy producers, such as cow genetics, feeding regimes, herd size, herd age and the volume of milk production appear to be effective at managing health issues in dairy cows to levels comparable to non-organic systems. Implementation of animal health plans that farmers can use as a management tool also have the potential to improve animal health and welfare and reduce the use of veterinarian medicines in these systems. However, there is still a need for scientifically assessed therapeutic treatment options. Furthermore, the evidence provided above suggests that the incidence and/or severity of some health issues are influenced by the level of animal productivity—something normally assumed to be lower on organic farms. This may change in the future if the demand for organic dairy products drives a rise in productivity. 

### 2.2. Sheep and Cattle

#### Gastrointestinal Nematodes

Gastrointestinal nematodes (GIN) are a major issue for livestock producers throughout the world, but are presumed to be particularly important for organically-reared animals due to restrictions on the use of anthelmintic treatments and increased access to the outdoors. Infection with GIN, particularly during the first-grazing season, leads to reduced productivity through parasite-induced inappetance, reduced efficiency of digestion and direct pathology in the gastro-intestinal tract [27,28]. In sheep, diarrhoea resulting from parasite infection can lead to breech-soiling, which in turn pre-disposes animals to blowfly strike.

In most livestock-producing regions, regular treatment with anthelmintics is required to remove existing worm burdens, reduce the build-up of the infective stages of the parasites on pasture and also as therapeutic treatments in individual animals displaying symptoms of parasitism [29]. It seems a reasonable assumption, therefore, that the impact of GIN would be greater in organic systems where anthelmintic treatments are restricted. This was supported in a study of five sheep farms by Cabaret *et al*. [30], who reported higher infection rates with GIN on organic than non-organic farms. In contrast, GIN egg production from calves did not differ between organic and non-organic dairy herds in Southern Scotland [28], and appeared to be lower in organic sheep flocks in a Canadian study [31]. However, the latter study was confounded as anthelmintic treatments were also withheld on the conventional farms. Organic farms use a variety of parasite control strategies, such as rotational/mixed grazing and the use of forage crops, instead of the prophylactic use of anthelmintics. For example, grazing juvenile animals on pastures not grazed by adults in the current or previous grazing seasons [27], alternate grazing with other species that do not share the same spectrum of parasites [32], or grazing animals on bioactive forages such as chicory [33]. The success of these strategies are likely to be significantly influenced by such factors as farm topography, climate and pasture productivity and this may partly explain the variable results reported in the literature. 

A wider range of parasitic helminths have been recorded on organic than non-organic farms. For example, post-mortem inspections of bull calves in Sweden demonstrated that infection with *Parafilaria bovicola* and *Dicrocoelium dendriticum* were more prevalent in organic animals [14], probably due to the fact that the vector for *P. bovicola* is commonly controlled by the use of fly-repellent ear tags on non-organic farms [14]. Lungworm (*Dictyocaulus viviparus*) was considered a major health concern for organic beef cattle in Denmark [34], as were the protozoan parasites *Eimeria* spp., the causative organism of coccidiosis.

Information comparing other health problems on organic and non-organic sheep or cattle farms is limited. However, an on-farm animal welfare monitoring scheme that included animal-based assessment criteria such as animal dirtiness, lameness and lesions, and culling age, was tested on 10 organic and 10 non-organic sheep farms [35]. This study concluded there was no difference in the welfare status of the animals between the organic and non-organic farms. As with dairy cattle, a proper and robust assessment of the impact of organic production systems on the health and welfare of sheep and beef cattle is difficult due to the potential confounding effects of differences in reporting statistics. 

A few studies have compared the incidence of gastrointestinal parasites in organic and non-organic dairy systems. Organic dairy farms had a higher parasitic burden than non-organic farms, but this difference between systems disappeared when season, grazing intensity and average milk production per cow was controlled for [16]. Organically reared dairy cattle also appeared to have a greater problem with parasitic infections than cattle in non-organic systems as assessed indirectly by outbreaks of diarrhoea in the first grazing season and lower weight gain during the grazing season relative to the previous season [26]. The predominant parasite management practices involve grazing management, such as turning calves out on pasture not grazed by cattle in the current or previous season and nutritional supplementation with concentrates and/or forages [26]. 

### 2.3. Pig and Poultry

As with sheep and beef cattle, endoparasites and ectoparasites are a major health problem for organic pig [36,37] and poultry [38] producers. Organic pig production, which is largely outdoors compared with non-organic indoor pig production, can result in higher parasitic infection levels and earlier acquisition of infections, including *Ascaris suum* and *Eimeria* spp. [31,38]. In a comparative study conducted in the Netherlands, the prevalence of coccidiosis and *Ascaris suum* in pigs was higher on organic than non-organic farms, but there was no difference in the prevalence of *Oesophagostomum* spp*.* and *Trichuris suis* among farm types [39]. Furthermore, the prevalence of *A. suum* was higher on free-range than conventional indoor farms, suggesting that prevalence of helminth infections is higher in pigs that have access to outdoor facilities regardless of their organic status.

In Austria, 75% of organic pig herds (84 farms) investigated recorded parasite eggs in faecal samples, with *A. suum* and *Oesophagostumum* spp*.* most prevalent. Coccidiosis and *T. suis* were also recorded, while lice and mange were detected in skin scrapings on 30% of farms. At slaughter, 50% of pigs displayed signs of milk spots on the liver, symptomatic of previous *A. suum* infection [37]. In organic pig herds in Denmark, 28% of weaners and 33% of fattening pigs were infected with *A. suum*, 4% of weaners and 13% of fattening pigs with *T. suis* and 20% of sows with *Oesophagostumum* spp., although no ectoparasites (e.g., scabies and lice) were found [40]. As these were not comparative studies, it is not possible to know the cause of these infections or how they compare to non-organically managed systems, but it is likely that access to outdoor environments is a risk factor for increased infections from helminths.

In a comparative study of poultry production systems, there was a higher prevalence of gastrointestinal helminth infections in free-range/organic and backyard systems, especially the incidence of *Heterakis gallinariu, Ascaridia galli*, *Capillaria obsignata* and *Capillaria anatis* [38]. Helminths were more common in the free-range/organic systems compared with battery cages where they were rarely identified, furthermore there no difference in the prevalence of these gastrointestinal helminths between free-range and organic farms [38]. Fossum *et al*. [41] reported a higher occurrence of parasitic diseases in laying hens reared in free-range compared with cage systems, where free-range systems included organic laying hens and birds that had access to outdoor pens and/or pastures. Few studies differentiate between free-range and organic housing systems in relation to the prevalence of parasitic infections, suggesting that access to the outdoors, regardless of organic status, is a risk factor for parasitic infection.

Other concerns for organic poultry systems include leg problems and increased infectious disease (e.g., Pasteurella). In one study, using fluctuating asymmetry (a measure of developmental instability) and conventional animal-based welfare indicators to assess organic and non-organically raised broiler chicken welfare, leg health appeared to be better in organic systems based on better hock condition and a longer latency-to-lie [42]. Reasons for differences between these production systems could include the use of different animal genotypes, age at slaughter, diet, stocking density and group size [42]. In a study of 18 organic egg producing flocks, mortality was predominantly caused by inappropriate behavioral patterns, disease and predatory attacks [43].

In summary, parasitic infections appear to be the major health issues associated with organic pig and poultry production. In addition, organic poultry systems may have increased risk of disease and predatory attacks. From the literature it is difficult to separate out the risk factors specifically associated with access to the outdoors from those of organic management systems. This is important as outdoor access itself conveys significant welfare advantages in the affective state and natural behavior domain. There may also be advantages to animal health associated with outdoor access and lower stocking densities in poultry such as a reduced incidence/severity of lameness. 

## 3. Affective State and Natural Behaviors

The term ‘affective state’ refers to a sentient animal’s emotional state and the presence of feelings. Procedures that cause pain, such as beak trimming in poultry, castration in beef cattle, sheep and pigs, tail docking in sheep and pigs and disbudding/dehorning in cattle are restricted under organic certification programmes. Although prohibiting such practices removes the pain of the procedures themselves, there is the increased potential for chronic suffering as a result. Procedures like beak trimming and tail docking are carried out to control feather pecking and tail biting; abnormal behaviors that are major welfare issues in themselves. Additional strategies are therefore required if these procedures are to be stopped. An inherent difficulty is that the cause of such negative behaviors is complex and not completely understood. As an example, feather pecking was studied in organic chicken farms, and the problem ranged from almost none to severe feather damage, despite the animals having ready access to outdoor runs [43]. 

Cannibalism was one of the main causes of death in laying hens in free-ranging systems in Sweden [41]. Hegelund *et al*. [43] found a variation in the prevalence of feather pecking amongst organically reared laying hens in Denmark with 6 out of 18 flocks showing little or no damage compared to 4 out of 18 flocks having indications of severe feather pecking. Risk factors for feather pecking in organically reared hens can include large group sizes, the absence of litter and the absence of daylight [44]. Understanding potential risk factors for feather pecking in organic systems allows the opportunity to develop management strategies to reduce these behaviors. 

When the severity of tail biting was assessed in pigs from five outdoor production systems (organic status unknown) at slaughter, the prevalence of tail-bitten pigs ranged from 14.1 to 20.1% [45]. In a review, the percentage of tail-bitten pigs observed either on-farm or at the abattoir was shown to range from extremely rare (0.1%) to very common (72%) [46]. This relative reduction in tail biting in outdoor-reared pigs is presumed to result from lower stocking densities and the ability to perform foraging/rooting behaviors, an increase in roughage in the diet and environmental enrichment [45]. This is supported by the fact that the incidence of tail biting decreased when pigs were provided with straw and increased with high stocking densities and when pigs were reared on slatted floors in indoor housing systems [47]. Despite reductions in the incidence of tail biting in pigs reared in outdoor based systems, it is obvious tail biting is still a serious issue [45]. Management strategies that could reduce these behaviors include better housing and the identification of genetically suitable populations best-suited to extensive rearing conditions [48]. 

The affective state of an animal may also be influenced by stockmanship and social stress among group housed animals [49]. Negative interactions with humans have been demonstrated to result in reduced productivity in animals [49,50] and can increase fearfulness [50]. An essential component of good farm management on organic and non-organic farms includes proper training of farm staff to manage and handle animals correctly to reduce the potential for fearfulness. Limited literature is available directly comparing stockmanship and the human-animal-relationship between organic and non-organic animal production systems, but good stockmanship is an essential component of any well managed farm. 

The natural-living concept of animal welfare infers that welfare is dependent on the degree that animals are able to perform ‘natural’ behaviors and live a ‘natural’ life [5]. Organic producers appear to consider natural living as important [48]. Organic farming standards emphasise that animals should be able to live as naturally as possible; this is usually accomplished by requiring that organically-raised animals are reared in loose housing systems, have adequate bedding, restricted areas of slatted floors, access to the outdoors and have reduced stocking densities compared with non-organic pasture and housing systems [51]. For example, organic systems require that hens have access to an outdoor run, which increases space allowance and the number of environmental stimuli that the animal is exposed to, increasing the opportunity for foraging behavior [52]. Increased space allowance and access and use of an outdoor run can reduce the risk of feather pecking and cannibalism in laying hens [44,53]. Organically raised animals are also fed diets that are higher in roughage, which improves their ability to perform natural feeding behaviors, such as foraging, rooting and chewing [54]. However, feed quality did appear to effect the motivation of dairy cows to feed as shown by a higher proportion of cows feeding during peak feeding time and a greater number of aggressive interactions at the feed-bunk between cows at peak feeding times on organic compared to non-organic managed farms [55]. Compared to some intensively managed non-organic systems, organically raised animals may therefore have more freedom to display ‘natural’ behaviors. However, extensive and intensive systems have welfare advantages and disadvantages and irrespective of the system, management is an essential component of maintaining good welfare [55,56].

In summary, organic systems can provide animals the increased opportunity to perform natural behaviors and organic standards result in a reduction in procedures that have the potential to negatively impact the affective state of animals, such as beak trimming and tail docking. Conversely, these standards can potentially impact the affective state of animals negatively if there is a higher incidence of abnormal behaviors such as feather peaking and tail biting. However, studies investigating risk factors associated with these behavioral problems can provide information potentially resulting in practical solutions to these issues. 

## 4. What Issues Need to be Addressed?

Studies published in peer-reviewed journals comparing the health and welfare of animals raised in organic versus non-organic farming systems, especially in species other than dairy cows, are limited. Therefore, this review primarily focuses on the dairy industry due to the limited number of studies published on other agricultural species reared in organic systems. As reviewed above, the majority of studies use databases of registered veterinary treatments; these are inherently limited or biased, in that cases of disease on organic farms where alternative remedies are predominantly being used will not tend to be recorded. For a more accurate picture of the health status of animals raised in different farming systems, detailed health records need to be collected. This would require farmers to keep adequate records of all health-related events, regardless of the production systems they employ [18]. This in turn requires that farmers are able to recognise health concerns and respond appropriately.

A lack of veterinarian input when animals are sick due to an adherence to organic philosophies of reduced reliance on conventional medical interventions is perceived by the authors to be a risk factor in ensuring high standards of animal health and welfare. This is also a concern that has arisen in the literature [17,51,57]. Nor is this risk confined to organic farmers; those employing non-organic methods also need to recognise and respond appropriately to animal health and welfare issues. Alternative treatments for sick animals can include frequent milking to reduce mastitis, heat liniments, homeopathy and herbal remedies. In a recent survey, 55% of organic farmers used alternative treatments for mastitis compared to 14% of conventional farmers [12]. The use of alternative therapies also varies significantly between organic farmers [9,58]. The major limitation in using alternative therapies is that their efficacy has generally not being appropriately tested. Furthermore, farmers may delay conventional treatments that are known to work, thereby unnecessarily prolonging the suffering of sick animals.

Another potential risk factor to timely treatment of animals with antibiotics when needed is the withdrawal period required before milk and meat products can be sold. EU Regulation 2092/91 states that the withdrawal period after use of allopathic veterinary medicine is double the legal period or, if not specified, 48 hours. The United States have stricter regulations than the EU and prohibit the use of antimicrobial drugs, but if these drugs are used then that animal loses its organic status [59]. The USDA organic regulations state that “the producer of organic livestock must not withhold medical treatment from a sick animal in an effort to preserve its organic status. All appropriate medications must be used to restore an animal to health when methods acceptable to organic production fail…” [59]. However, we speculate that the economic loss due to withdrawal periods or loss of organic status of an animal may cause some farmers to delay treatment with antibiotics until they think there is no alternative but to administer these treatments, thereby potentially prolonging the unnecessary suffering of sick animals. 

Even if the overall disease incidence is similar or lower on organic farms, there may still be welfare issues for individual animals which are not being treated appropriately or in a timely manner. It would be advantageous for the organic industry to have acceptable alternative therapies that have been empirically evaluated as to their effectiveness.

An alternative approach that could minimise potential health problems on organic farms is the selection of animals that are more suited to low- or non-chemical input systems. Desirable traits for organic systems include resistant to diseases, reduced productivity, improved natural behavior and coping in extensive/outdoor situations; an obvious example is the use of *Bos indicus* cattle in extensive grazing systems. 

Management of organic farms is based on a combination of general organic principles and more detailed rules set out by certification bodies. Organic standards and regulations can differ among countries and regulations within countries have evolved over time, such as those governing antibiotic use. This needs to be taken into account when comparing the incidence of disease or animal-based welfare assessments in studies published over several years and in different countries. Even among producers who follow the same organic guidelines there can be a large variation in the incidence of disease and the welfare of animals. A large range in the incidence of clinical mastitis [9], lameness [22], and hock lesions [23] was observed among organic farms. Differences in animal health and welfare among farms may be directly related to how these different farms are managed and the motivation behind a producer converting to organic farming practices. For example, dairy farms that converted to organic practices before 1990 had the lowest treatment frequency and the lowest calculated bulk tank SCC over an 8 year period compared to farms that converted after 1994 or those managed non-organically [15]. One possible explanation for this is that farms that converted after 1994 may have been motivated more by financial subsidies from producing organic products and not necessarily due to their belief in the philosophies behind organic agriculture [15]. This could become an increasing problem as the demand for organic products increase. These differences in animal health problems among farms suggest that organic certification, although having a beneficial impact on some health measures does not always ensure cow welfare [23]. Welfare may be more closely linked to good management than the principles of organic farm management. It is therefore important for the organic industry to capitalise on the knowledge and practices of their best performing farmers. To this end the implementation of health plans that can be used as management tools by farmers show promise as a means to achieve and maintain a high health status of animals reared on organic farms [21]. 

## 5. Conclusions

The primary welfare risk identified in the literature in organic management systems appears to be related to biological function, specifically animal health. The other main domains of animal welfare including affective state and the ability to perform natural behaviors in organics systems have not been well studied. These domains however appear to be the ones that resonate most with consumers when they consider welfare in organic systems due to assumptions of increased outdoor access, space and ability to perform natural behaviors. This is a potential area for the organic livestock farming industry that needs to be quantified as it could be a documented benefit which would compensate for other deficiencies. 

Organic farms primarily use management practices and alternative remedies to manage the health of their animals although in many studies animal health is similar between organic and non-organically run herds. This equivalence should be treated with caution however as it may be due to under-recording of disease incidence on organic farms. The variation in disease incidence between farms and the apparent higher incidence of some health problems on newly converted organic farms suggests that management skills and experience make a significant contribution to animal welfare. One concern of organic systems from an animal welfare standpoint is that if an animal becomes sick, conventional medicines such as antibiotics may be withheld until alternative remedies have failed, which could result in prolonged suffering of that animal. A more prescriptive point of intervention may need to be developed to assure good welfare in this situation. Alternative, organically acceptable treatments also need to be scientifically evaluated so that organic farmers have tools that they can use and know when to use an alternative treatment and when to use a conventional one to safeguard the animal’s welfare. On-farm animal-based welfare assessment audits to assure the welfare of animals on organically managed farms need to be developed to confirm that good welfare standards are being achieved. 
